# Aicardi syndrome: The importance of an ophthalmologist in its diagnosis

**DOI:** 10.4103/0301-4738.49403

**Published:** 2009

**Authors:** Parag K Shah, V Narendran, N Kalpana

**Affiliations:** Pediatric Retina Department, Aravind Eye Hospital and Postgraduate Institute of Ophthalmology, Coimbatore, Tamilnadu, India

**Keywords:** Aicardi syndrome, chorioretinal lacunae, fundus findings, ophthalmologist

## Abstract

Aicardi syndrome is a rare genetic disorder. The salient features of this syndrome include agenesis of corpus callosum, chorioretinal lacunae and infantile spasms. Of these three, chorioretinal lacunae is the most constant feature present. This case highlights the importance of fundus findings by an ophthalmologist in making the diagnosis of this rare syndrome.

Aicardi syndrome is a rare congenital disorder that was first described by a French neurologist, Jean Aicardi in 1965.[1] It is characterized by a classic triad of agenesis or hypogenesis of the corpus callosum, infantile spasms and chorioretinal lacunae. It is a X-linked disorder and is seen almost exclusively in females. We report a case of this rare disorder.

## Case Report

A three-month-old female child was referred to our hospital for evaluation of apparent blindness. There was history of frequent episodes of flexor spasms with twitching of both eyes since one and a half months of age. Each episode recurred nearly five to six times per day associated with drooling of saliva. According to the parents, the child never turned towards light or sound. She was a second child born to a non-consanguineous marriage. It was a full-term normal delivery. There was no history of jaundice, fever or convulsion in the immediate neonatal period.

On examination, there was global developmental delay. The head circumference was in 10^th^ percentile. On ophthalmic examination the child was neither responding to nor following bright light. Both the pupils were very sluggish in reaction. Anterior segments of both eyes were normal. The vitreous was clear and normal in both eyes. Fundus examination revealed bilateral optic nerve colobomas with variable-sized discrete dome-shaped loci of pale areas with sharp borders (chorioretinal lacunae) just nasal to the optic discs [Fig. 1a, 1b]. The fovea was spared in both the eyes.

**Figure 1a:**
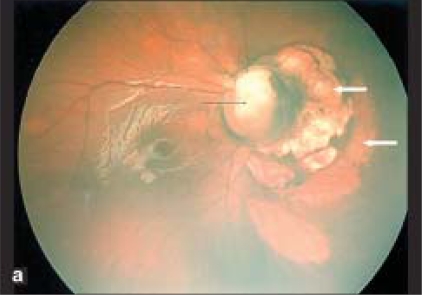
Retcam photo of right eye shows optic disc coloboma (black arrow) and dome shaped loci of pale areas with sharp borders nasal to the optic disc suggestive of chorio retinal lacunae (white arrows).

**Figure 1b:**
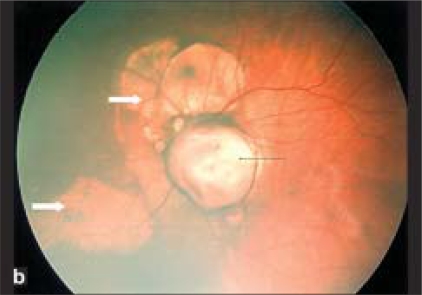
Retcam photo to left eye showing also showing optic disc coloboma (black arrow) and chorio retinal lacunae nasal to optic disc (white arrow).

**Figure 2:**
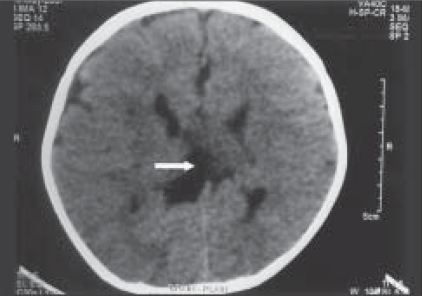
Axial CT brain showing parallelism of ventricles with a small inters hemispheric cyst (white arrow) and no impression of splenium suggestive of hypogenesis of corpus callosum.

As the child had seizures, electroencephalography (EEG) was done by the neurologist. EEG showed burst of spike, polyspike, sharp and slow-wave complexes with suppression in between, predominantly over the right hemisphere. Bilateral synchronous and symmetrical discharges were also present along with slow waves of high amplitudes that were seen more on the right hemisphere. Thus, it showed burst suppression and hypsarrhythmia pattern. Computed tomography (CT) scan of the child revealed hypogenesis of the corpus callosum with small inter-hemispheric cyst [Fig. 2].

She was on anti-epileptic medications and subcutaneous injections of adrenocorticotropic hormone (ACTH) and was having good seizure control.

## Discussion

Aicardi syndrome is a rare X-linked dominant genetic disorder. The syndrome is almost exclusively seen in females with early embryonic lethality in hemizygous males.[2] The diagnosis of this disorder is mainly based upon the classic triad of infantile spasms, chorioretinal lacunae and corpus callosum agenesis. Infantile spasms typically start in early childhood. Dissociated burst suppression or burst suppression pattern appearing asymmetrically in either cerebral hemisphere is a characteristic EEG finding in this syndrome,[3] which was seen in our case. Developmental delay is generally profound involving both motor and language skills. Chorioretinal lacunae are well-defined, multifocal pale areas with minimally pigmented borders, and they are usually clustered around the optic disc. They are peculiar punched out areas of choroidal and retinal pigment epithelium (RPE) atrophy. Other ocular abnormalities reported are optic nerve colobomas, which was seen in our case, optic nerve hypoplasia, optic disc pigmentation, microphthalmos, retrobulbar cyst, pseudoglioma, retinal detachment, macular scars, cataract, pupillary membrane remnants, iris synechiae and iris coloboma.[4] Good visual function is seen if fovea is spared of the chorioretinal lacunae, as seen in our case. Costovertebral malformations such as hemivertebrae, fusion of vertebrae, kyphoscoliosis, absent or malformed ribs, and occasionally cleft lip and palate may also be associated with Aicardi syndrome.[2] However, none of these findings were detected in our case. Most of the Aicardi syndrome cases die at an early age due to aspiration pneumonitis. But some do live up to the adolescent years and even into their twenties.[5] ACTH, prednisolone, valproic acid and clonazepam have been used with variable success.[6]

Of late a larger spectrum of the disease has been recognized. In 1998, Aicardi[7] reported that corpus callosum agenesis is not the hallmark of the disease and is not even necessary for diagnosis if other cerebral pathologies, such as cysts, are present. Fundus examination has gained increasing importance when Aicardi[6] reported in 2005 that no feature except chorioretinal lacunae is constant. Hyogenesis of the corpus callosum and infantile spasm can be seen in a number of other disorders but in diagnosing Aicardi syndrome the ophthalmologist can play a pivotal role in looking for chorioretinal lacunae and clinching the diagnosis, as in our case.

Thus, in conclusion, Aicardi syndrome should be kept in mind while investigating a female child with recurrent seizures in early childhood, and fundus examination by an ophthalmologist can be of immense help in the diagnosis of this rare genetic disorder.
